# New Treatment for Varix

**Published:** 1852-09

**Authors:** 


*New Treatment for Varix.*

In the *Revue Medico Chirurgicale*, M. Durant suggests a new method of treating Varix. It consists in applying to the tumor or varix three successive layers of collodion, the whole covered with a piece of silk, moistened also with collodion. This little apparatus is simple, easily applied, and ought to be removed as often as once in eight or ten days. M. Durant requests the profession to try this agent, in order to determine its value in these affections. J.

